# Fast whole brain relaxometry with Magnetic Resonance Spin TomogrAphy in Time-domain (MR-STAT) at 3 T: a retrospective cohort study

**DOI:** 10.1007/s10334-025-01237-3

**Published:** 2025-03-04

**Authors:** Martin B. Schilder, Stefano Mandija, Sarah M. Jacobs, Jordi P. D. Kleinloog, Hanna Liu, Oscar van der Heide, Beyza Köktaş, Federico D’Agata, Vera C. W. Keil, Evert-Jan P. A. Vonken, Jan Willem Dankbaar, Jeroen Hendrikse, Tom J. Snijders, Cornelis A. T. van den Berg, Anja G. van der Kolk, Alessandro Sbrizzi

**Affiliations:** 1 Computational Imaging Group for MR Therapy and Diagnostics, UMC Utrecht, Utrecht, Netherlands; 2 Department of Radiology and Nuclear Medicine, UMC Utrecht, Heidelberglaan 100, 3584 CX Utrecht, The Netherlands; 3 Department of Neurosciences, University of Turin, Turin, Italy; 4 Department of Radiology, Amsterdam UMC, Amsterdam, Netherlands; 5 Department of Radiotherapy, UMC Utrecht, Utrecht, Netherlands; 6 Department of Neurology & Neurosurgery, Brain Center, UMC Utrecht, Utrecht, Netherlands; 7 Department of Medical Imaging, Radboud UMC, Nijmegen, Netherlands

**Keywords:** Magnetic resonance imaging, Relaxometry, Neuroimaging, Quantitative magnetic resonance imaging

## Abstract

**Objective:**

To report *T*_1_/*T*_2_-values of normal and normal appearing brain tissues (NBTs, healthy volunteers; NABTs, patients) acquired with a whole-brain 5-minute Magnetic Resonance Spin TomogrAphy in Time-domain (MR-STAT) protocol, and to explore relaxometry behavior in a brain tumor and in a multiple sclerosis patient.

**Methods:**

MR-STAT was acquired in 49 participants (39 patients with neurological pathologies, age range: 21–79 years) at 3 T. Mean *T*_1_/*T*_2_-values were computed for: normal and normal appearing grey matter (NGM/NAGM)/white matter (NWM/NAWM)/thalamus/putamen/caudate nucleus (CN)/globus pallidus (GP). Differences between sex, brain lobes, and left/right were assessed. The age-dependency of *T*_1_/*T*_2_-values in N(A)BTs was investigated. Relaxometry analysis was performed in two clinical case examples.

**Results:**

Mean (standard deviation) *T*_1_/*T*_2_-values were measured in N(A)GM = 1086(73)/74(9) ms; N(A)WM = 658(24)/48(3) ms; thalamus = 783(51)/42(4) ms; putamen = 863(40)/46(3) ms; CN = 1042(97)/63(9) ms; GP = 652(36)/36(3) ms. Differences between sex were not significant. *T*_1_/*T*_2_-values between the left/right parietal lobe and the left/right temporal lobe were significantly different. The quadratic age-dependency of *T*_1_-values in the CN (*p* = 0.00039) and GP (*p* = 0.00037), and of *T*_2_-values in the thalamus (*p* = 0.00044) and GP (*p* = 0.003) were significant. Pathological tissues could be discerned from NABTs using *T*_1_/*T*_2_-values.

**Discussion:**

*T*_1_/*T*_2_-values and data trends agree with literature, supporting the validity of MR-STAT as a clinical option for fast relaxometry despite the relatively low number of subjects in the study. Future work should aim to include healthy participants of a wider age-range and to include B_1_-field corrections.

**Supplementary Information:**

The online version contains supplementary material available at 10.1007/s10334-025-01237-3.

## Introduction

Through magnetic resonance (MR) relaxometry, the magnetic properties *T*_1_ and *T*_2_ of human tissue can be measured [1]. Quantitative data potentially allows for unequivocal tissue characterization and might provide quantitative imaging biomarkers when compared to conventional, qualitative assessments. For instance, it has previously been shown that differences between Parkinson’s disease patients and controls can be found based on relaxometry properties of cortical grey matter [2].

Over the past 20 years, brain relaxometry has undergone major developments. At the start of the twenty-first century, techniques like inversion recovery true fast imaging with steady state precession (TrueFISP) [3] and quantification of relaxation times and proton density by twin-echo saturation-recovery turbo-field echo (QRAPTEST) [4] were introduced. In more recent years, faster quantitative multiparametric magnetic resonance imaging (MRI) frameworks have been developed, such as MR Fingerprinting (MRF) [5–8], STrategically Acquired Gradient Echo (STAGE) [9–11], synthetic MRI (syMRI) [12, 13], MR Multitasking [14–16] and MR Spin TomogrAphy in Time-domain (MR-STAT) [1, 17, 18]. In this work, we focus on the latter technique.

MR-STAT is a multiparametric MRI technique that simultaneously maps *T*_1_, *T*_2_ and proton density (PD) from a 5-min acquisition [1, 18, 19]. The quantitative parameter maps are reconstructed by directly fitting a volumetric signal model to a transient-state time-domain signal. During the fitting, spatial localization of the signal and quantification of *T*_1_, *T*_2_ and PD are done simultaneously. The reconstruction is performed by numerically solving a large-scale non-linear problem. Recent advances in MR-STAT have included the clinical demonstration of synthetic MR-STAT images [20], accelerated parameter map reconstructions [21] and a 3D MR-STAT protocol for high-resolution knee imaging [22] and brain imaging [23]_._ Furthermore, good repeatability of MR-STAT has been demonstrated in gel phantoms [17, 24] and in vivo [23]. However, a study that reports relaxation times of normal brain tissues (NBT) in healthy volunteers and normal appearing brain tissues (NABTs) in patients of a cohort of relevant size using MR-STAT has not been presented.

The aim of this observational study is, therefore, to report *T*_1_ and *T*_2_ relaxation times of normal and normal appearing grey matter (NGM, in healthy volunteers/NAGM, in patients), normal and normal appearing white matter (NWM, in healthy volunteers/NAWM, in patients) and subcortical brain regions (thalamus, putamen, caudate nucleus and globus pallidus) measured at 3 T with MR-STAT in 49 subjects. In addition, we investigate: (a) differences in relaxation times between male and female participants to preliminarily assess bias towards sex; (b) differences between brain lobes to assess systematic biases; (c) left versus right differences within brain lobes to assess left–right biases; and (d) the relation between *T*_1_- and *T*_2_-values of N(A)BTs and age to find whether MR-STAT data confirms quadratic trends in *T*_1_ and *T*_2_ of N(A)GM, N(A)WM and subcortical brain regions that were described earlier in literature [25, 26]. If confirmed, values from NABTs could be used to preliminarily extend work on young healthy volunteers to elderly healthy volunteers. Lastly, our study concludes with two example cases of MR-STAT relaxometry in neurological brain disorders, namely a primary brain tumor and a multiple sclerosis (MS) lesion.

## Methods

### Study participants

Between October 2019 and October 2021, a total of 50 adult (≥ 18 years) subjects were included in the first MR-STAT clinical study [20]. The subjects were either healthy (*n* = 10), defined as lacking history of neurological disease and no imaging findings to suspect otherwise, or had the clinical diagnosis of primary brain tumor (*n* = 11), ischemic stroke (*n* = 10), epilepsy (*n* = 10) or MS (*n* = 9). The study was registered in the international clinical trials registry platform with number NL8437 (https://trialsearch.who.int/Trial2.aspx?TrialID=NL-OMON26690) and was conducted according to the guidelines described in the Declaration of Helsinki, and approved by the Institutional Review Board (NL69544.041.19, METC 19/282). Written informed consent was given by all participants prior to scanning. Example images of the quantitative parameter maps (*T*_1_, *T*_2_, PD) and conventional and synthetic contrast weighted images including all 30 slices from a healthy participant and a patient of each group are in a publicly available repository (https://gitlab.com/asbrizzi/mr-stat-synthetic-images).

For the current study, all patients were retrospectively included in this analysis from the above-mentioned trial. However, one patient had a large bilateral stroke and NABTs could thus insufficiently be identified. This patient was retrospectively excluded from our analysis since the primary focus of this study is to report on relaxation times of N(A)BTs. In addition, if an epilepsy patient had a (benign) tumor as underlying cause of the symptoms, this patient was transferred to the tumor group, which was the case for six epilepsy patients. This led to a study population consisting of 10 healthy volunteers, 17 tumor patients, 9 stroke patients, 4 epilepsy patients and 9 MS patients. A summary of the study population can be found in Table 1.

**Table 1:** Summary of patient population

Group	*N*	Male/female	Mean age in years (standard deviation)
Total population	49	27/22	44.2 (16.1)
Healthy volunteers	10	6/4	24.2 (2.7)
Tumor patients	17	10/7	47.9 (14.5)
Stroke patients	9	4/5	51.8 (14.5)
Epilepsy patients	4	3/1	50.8 (15.0)
Multiple sclerosis patients	9	4/5	45.9 (12.9)

### Acquisition and processing

MRI acquisitions were performed on an Ingenia scanner (Philips Healthcare, Best, the Netherlands) at 3 T using a clinical 15 channel receiver head coil (Philips Healthcare, Best, the Netherlands). Conventional contrast weighted scans (T_1_w, T_2_w, PDw, FLAIR) and MR-STAT were acquired. The exact sequence parameters are listed in Table 2. The MR-STAT sequence consisted of multiple Cartesian-encoded 2D slices. Each slice was sequentially acquired with a gradient-spoiled gradient echo scheme and a slowly varying flip angle with amplitude between 0° and 90°. The flip angle train is shown in Supplementary Material A. Each RF train was preceded by a non-selective inversion pulse [17]. In total, 30 axial slices with 1 × 1 × 3 mm^3^ resolution and an interslice gap of 1.5 mm were acquired. Scan time for the MR-STAT protocol was roughly 5 min. The reconstruction was performed according to methods presented in Liu et al*.*, consisting of an alternating direction method of multipliers [21]. The offline reconstruction time was approximately 2 min per slice.

**Table 2:** Acquisition parameters of MR-STAT imaging and conventional imaging. An axial scan orientation was used

Imaging parameters	MR-STAT Spoiled GRE	*T﻿*_1_w SE	*T*_2_w SE	PDw TSE	FLAIR TSE
FOV	224 × 224 × 133.5 mm^3^				
Spatial resolution	1 × 1 × 3 mm^3^				
Gap	1.5 mm				
Slices	30				
TR (ms)	8.9	451	3400	2800	10,000
TI (ms)	–	–	–	–	2800
TE (ms)	4.7	14	80	20	120
Flip angle	Variable	70	90	90	90
TSE factor	–	–	15	14	24
Scan time	5:11 min	3:16 min	1:44 min	3:40 min	1:41 min

*MR-STAT:* Magnetic Resonance Spin TomogrAphy in Time-domain; *PD:* Proton Density weighted; *FLAIR:* Fluid Attenuated Inversion Recovery; *GRE:* gradient echo; *SE*: spin echo; *TSE:* turbo spin echo; *FOV:* field of view; *TR:* repetition time; *TI:* inversion time; *TE:* echo time

Synthetic T_1_w images were generated using an analytical signal model as described in more detail in Kleinloog et al. [20]. Automatic brain tissue segmentations were performed on the synthetic T_1_w images using the software Vol2brain [27] for the following cerebral brain structures: cortical grey matter, cortical white matter, thalamus, putamen, caudate nucleus, and globus pallidus. Due to the limited spatial resolution in the craniocaudal direction, the cerebellum was excluded from the analysis. Vol2brain also provided masks for each brain lobe and a hemisphere mask. For all patients, lesions were manually segmented using FSL software [28] and were subtracted from all other masks. This lead to masks for NABTs, that we defined as patients’ tissues without visible lesions.

### Preliminary test: relaxation times of NBTs and NABTs

Twelve out of seventeen tumor patients had received radiotherapy prior to this study. To test if the *T*_1_- and *T*_2_-values of NAGM and NAWM differed significantly between the tumor patient group that did receive radiotherapy and the tumor patient group that did not, a Mann–Whitney *U* test was performed with a significance level of 0.05. Bonferroni correction was not performed to prevent making type II errors. Next, the mean *T*_1_- and *T*_2_-values were computed for the N(A)GM, N(A)WM and for the normal and normal appearing thalamus, putamen, caudate nucleus and globus pallidus. These values were reported for both the healthy participants and the complete study population.

### Statistical analysis

All statistical tests were performed in MATLAB 2019A (MathWorks, Natick, MA, USA). For the sex-related differences and comparison between brain lobes (tests 1–3), only data from healthy volunteers was used. However, to perform meaningful statistical tests with respect to dependency of *T*_1_- and *T*_2_-values on age (test 4), a wider age-interval was needed. Therefore, data from NBTs from healthy volunteers and data from NABTs from patients were bundled in this part of the analysis. For each of the tests, a significance level of 0.05 was used before Bonferroni correction was applied. The corresponding significance level is mentioned for each of the tests.

#### Test 1: Sex-related differences

A Mann–Whitney U test was used to test if there were sex-related differences within the study population between the *T*_1_-values and *T*_2_-values of NGM and NWM. Since this test included a small sample (male participants: *n* = 6; female participants: *n* = 4), no meaningful assumption about the underlying distribution (e.g. a normal distribution) could be made. Therefore, a non-parametrical test with Bonferroni correction was applied. The corresponding significance level was 0.0125. To ensure age was not a confounding factor, another Mann–Whitney *U* test was performed to test if there were significant differences in age between the male and female group.

#### Test 2: Differences between brain lobes

The *T*_1_-values of NGM and NWM from the frontal, parietal, occipital and temporal brain lobes were compared. Here, data from both the left and right hemispheres were bundled. A Wilcoxon signed rank with Bonferroni correction and a corresponding significance level of 0.0021 was used. As B_1_-effects confound the *T*_2_-values, we did not test for significance in *T*_2_-values.

#### Test 3: Left–right brain lobe differences

In this test, *T*_1_- and *T*_2_-values of NGM and NWM of the left versus right brain lobes were compared to test whether differences were statistically significant. For this comparison, a Wilcoxon signed rank test with Bonferroni correction and a corresponding significance level of 0.0031 was used.

#### Test 4: Dependency of relaxation times on age

A quadratic curve was fit to *T*_1_- and *T*_2_-values of N(A)GM, N(A)WM and normal (appearing) thalamus, putamen, caudate nucleus and globus pallidus. The function that was fit was $T\left(\text{age}\right)=a \times {\text{age}}^{2}+b\times \text{age}+c$. The use of a quadratic curve to fit *T*_1_ and *T*_2_ of N(A)GM, N(A)WM and subcortical brain regions was described earlier in literature [25, 26]. An *F*-test with Bonferroni correction was performed to determine if a quadratic function fitted the data better than a linear function, with a corresponding significance level of 0.0042.

### Case examples

To further demonstrate the potential of quantitative MRI using MR-STAT, this paper was concluded with two case examples, namely for a tumor patient and an MS patient. A case example for a stroke patient was not provided since the stroke events for the included patients occurred several months or even years prior to data acquisition. Regarding the epilepsy group, since the causes of the epilepsy were either no longer visible (because of previous surgery), or consisted of sclerosis of the hippocampus (which was not part of this analysis), no example case from the epilepsy group was provided.

#### Case example 1: Tumor patient

The first example case describes the *T*_1_- and *T*_2_-findings of a tumor patient. The imaging findings from this patient were selected because no treatment had been given at the time of scanning (and thus the quantitative values had not been influenced by treatment effects), and because the lesion was well delineable. The NAGM, NAWM and lesions were segmented according to methods described above. As a contrast agent was not administered, the enhancement status of the tumor is unknown.

#### Case example 2: Multiple sclerosis patient

The second case report concerns a patient with MS. The NAGM, NAWM and lesions were segmented according to methods described above. An analysis of *T*_1_- and *T*_2_-values was performed on one lesion that clearly shows as a T_1_ black hole. Since no contrast agent was administered, the enhancement status of the lesion is unknown.

## Results

### Preliminary test: relaxation times of NBTs and NABTs

No statistically significant differences were found between *T*_1_ of NAGM and NAWM and *T*_2_ of NAGM and NAWM of tumor patients that had undergone radiotherapy treatment prior to this study, versus tumor patients that had not (*p* = 0.6787, *p* = 0.9530, *p* = 0.6787, *p* = 0.8591, respectively). However, because the group had been treated with radiotherapy, the groups remained split for further analysis.

The mean *T*_1_- and *T*_2_-values over the segmented brain regions are reported in Table 3 for the healthy participants and for the population as a whole. A further breakdown per patient group was provided in Supplementary Material B.

**Table 3:** Mean and standard deviations of *T*_1_ and *T*_2_ of NBTs from healthy volunteers and N(A)BTs from the mixed clinical population and literature

	Mean *T*_1_ (± SD) [ms]			Mean* T*_2_ (± SD) [ms]		
Brain region	Healthy volunteers (*n* = 10)	Total population (*n* = 49)	Literature ranges [7, 8, 11, 13, 14, 25, 26, 29–45]	Healthy volunteers (*n* = 10)	Total population (*n* = 49)	Literature ranges [7, 8, 11, 13, 14, 25, 26, 29–45]
N(A)GM	1136 (82)	1086 (73)	790–1618	77 (12)	74 (9)	53–130
N(A)WM	657 (18)	658 (24)	620–954	48 (3)	49 (3)	29–120
Thalamus	771 (49)	783 (51)	860–1262^a^	40 (3)	42 (4)	60–74^a^
Putamen	878 (47)	863 (40)	920–1328^a^	46 (4)	46 (3)	49–64^a^
Caudate nucleus	1028 (69)	1042 (97)	960–1379^a^	61 (8)	63 (9)	59–73^a^
Globus pallidus	664 (24)	652 (36)	800–1055^a^	36 (2)	36 (3)	–

^a^ Mean *T*_1_- or *T*_2_-values were not always explicitly provided in early published literature. Thus, values from individual study participants were estimated from the figures in the publication

### Statistical analysis

#### Test 1: Sex-related differences

**Fig. 1:**
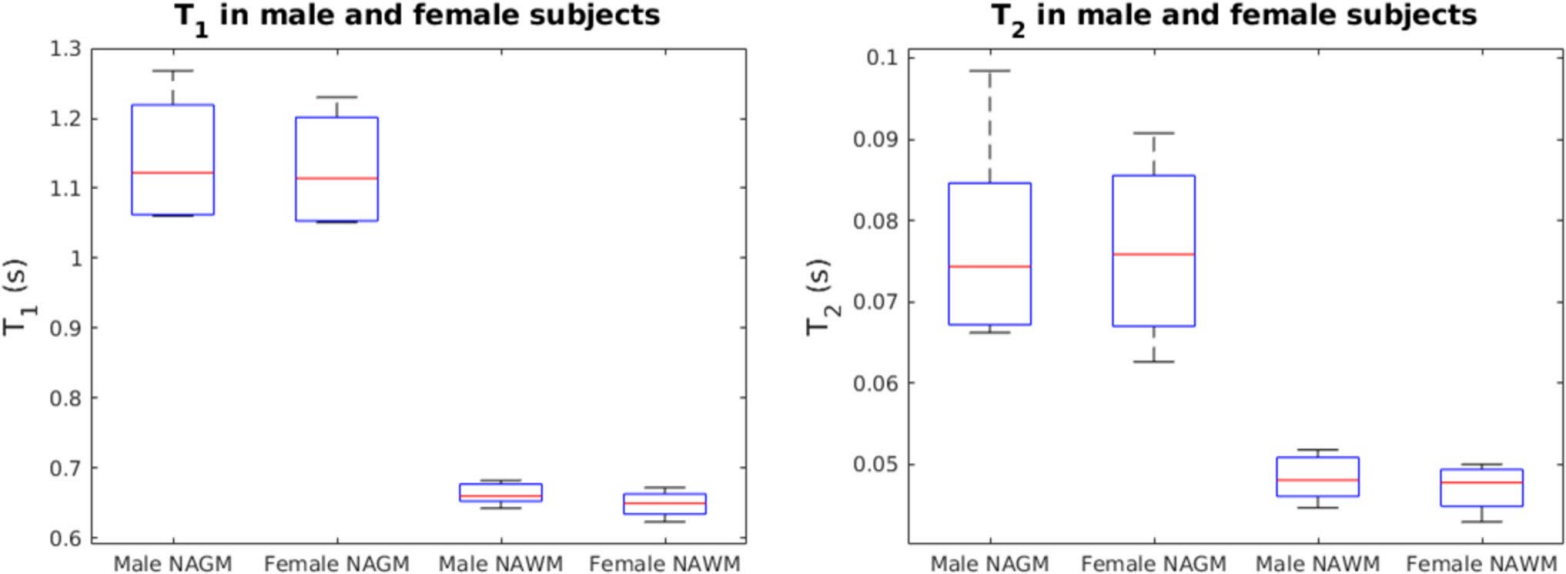
Boxplots comparing *T*_1_- and *T*_2_-values of NGM and NWM between male and female subjects. The mean age of the male population was 24 (± 1.8) years. The mean age of the female population was 24.5 (± 4.0) years. Differences in age between the group were not statistically significant (*p* = 0.8762). Age was thus comparable between the groups. The red line indicated the median, the box indicates the upper quartile and the lower quartile and the whiskers indicate one standard deviation above and below the mean

Differences in age between the group were not statistically significant (*p* = 0.8762). Age was thus comparable between the groups. Figure 1 shows boxplots of *T*_1_ and *T*_2_ of NGM and NWM of the male and female healthy participants. No statistically significant differences were found between the *T*_1_ and *T*_2_ of NGM and NWM of male and female subjects (*p* = 0.580, *p* = 0.330, *p* = 0.944, *p* = 0.350).

#### Test 2: Differences between brain lobes

**Fig. 2:**
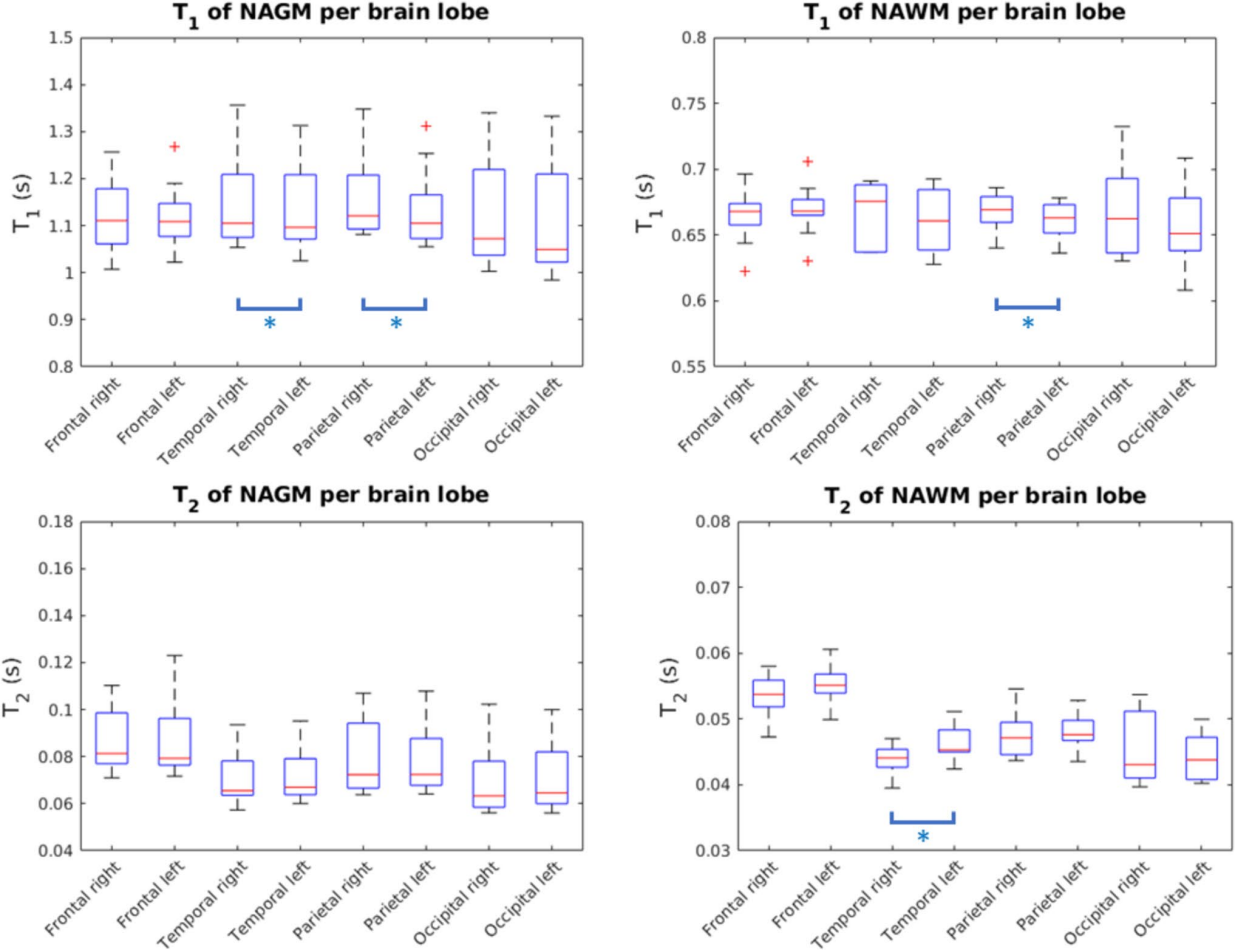
Boxplots visualizing *T*_1_- and *T*_2_-values of NGM and NWM per brain lobe. Only data from healthy volunteers was used. There were no significant results from test 2. Blue bars: single asterisks indicates significant differences between lobes from the left versus right hemisphere. All other differences were not significant (test 3)

Figure 2 shows the boxplots of *T*_1_ and *T*_2_ of NGM and NWM for each brain lobe. In the comparison between brain lobes, for the *T*_1_ of NGM and NWM, no significant differences between brain lobes were found. A table with *p*-values for all comparisons can be found in Supplementary Material C.

#### Test 3: Left–right brain lobe differences

Statistically significant differences were found between the *T*_1_ of NGM of the left versus the right temporal lobe, between the *T*_1_ of NGM of the left versus right parietal lobe, between the *T*_1_ of NWM of the left versus right parietal lobe and between the *T*_2_ of NWM of the left versus right temporal lobe. All other differences were not significant. Statistically significant differences between hemispheres were thus limited to the parietal lobe and in the temporal lobe. A table with *p*-values was provided in Supplementary Material D.

A table with *T*_1_- and *T*_2_-values per brain lobe was provided in Supplementary Material E.

#### Test 4: Dependency of relaxation times on age

**Fig. 3:**
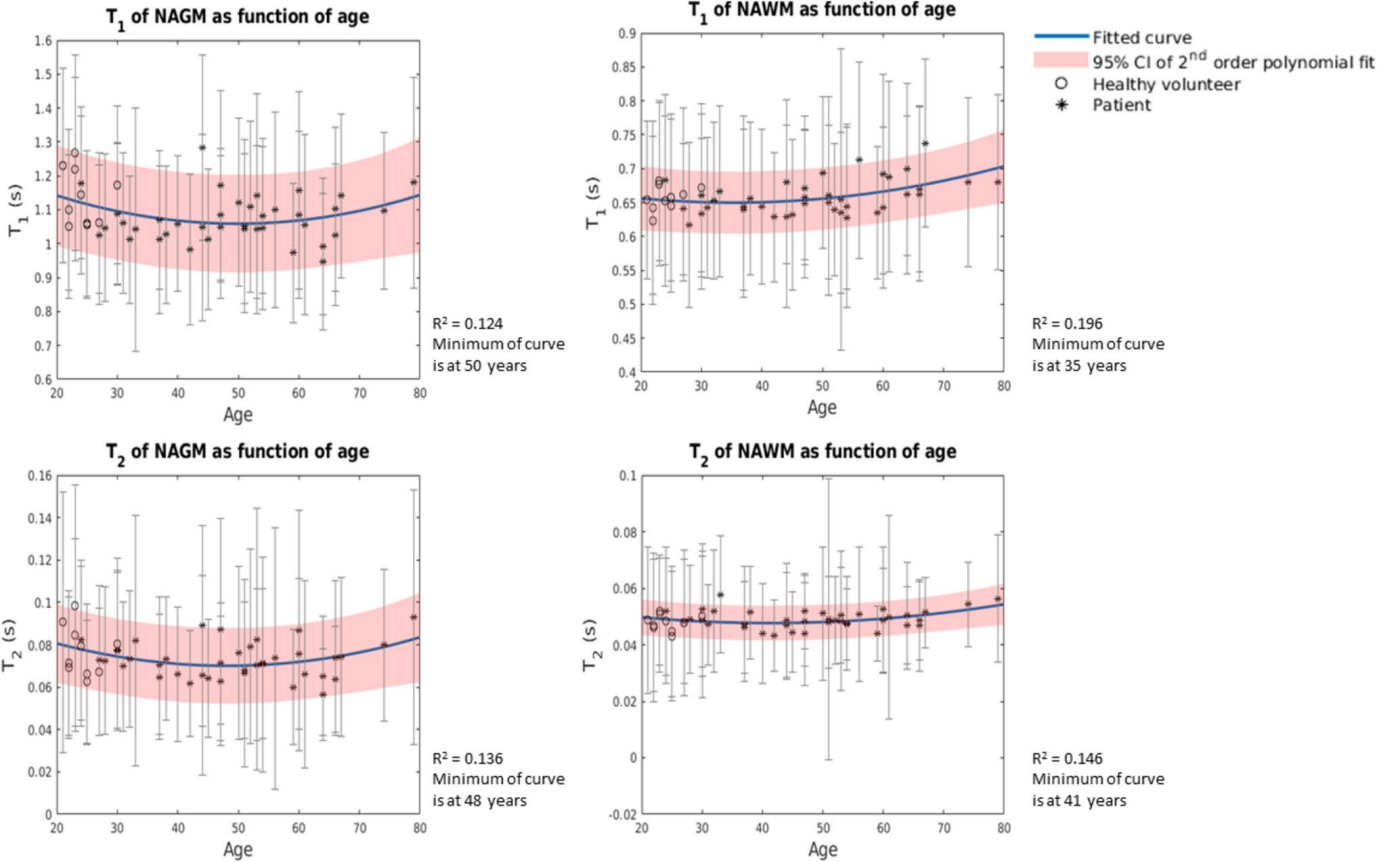
Fitted quadratic curves for *T*_1_ and *T*_2_ of N(A)GM and N(A)WM. The blue curve indicates the fitted model. The red lines indicate the 95% confidence interval of the fit. The bars in the plot through the datapoints indicate the standard deviation of *T*_1_ and *T*_2_ within N(A)GM and N(A)WM

In Fig. 3, the quadratic fits for age and *T*_1_ and *T*_2_ of N(A)GM and N(A)WM are shown. For the *T*_1_ of N(A)GM, the coefficient of determination resulted to be R^2^ = 0.124 (*p* = 0.048) and for the *T*_1_ of N(A)WM, R^2^ = 0.196 (*p* = 0.007). We found R^2^ = 0.136 (*p* = 0.035) for the *T*_2_ of N(A)GM and R^2^ = 0.146 (*p* = 0.027) for the *T*_2_ of N(A)WM. The minima of the four curves were found at 50 years, 35 years, 48 years and 41 years, respectively. The quadratic fits for the *T*_1_ of the caudate nucleus (*p* < 0.001) and the globus pallidus (*p* < 0.001) and the *T*_2_ of the thalamus (*p* < 0.001) and the globus pallidus (*p* = 0.003) were significant. All other quadratic fits were not statistically significant. An overview of the exact coefficients of determination, *p*-values and the coefficients of the quadratic fit for the N(A)BTs can be found in Supplementary Material F. Overall, we observed a quadratic trend, which is in line with earlier literature [6, 25, 26].

### Case examples

#### Case example 1: Anaplastic glioma

**Fig. 4:**
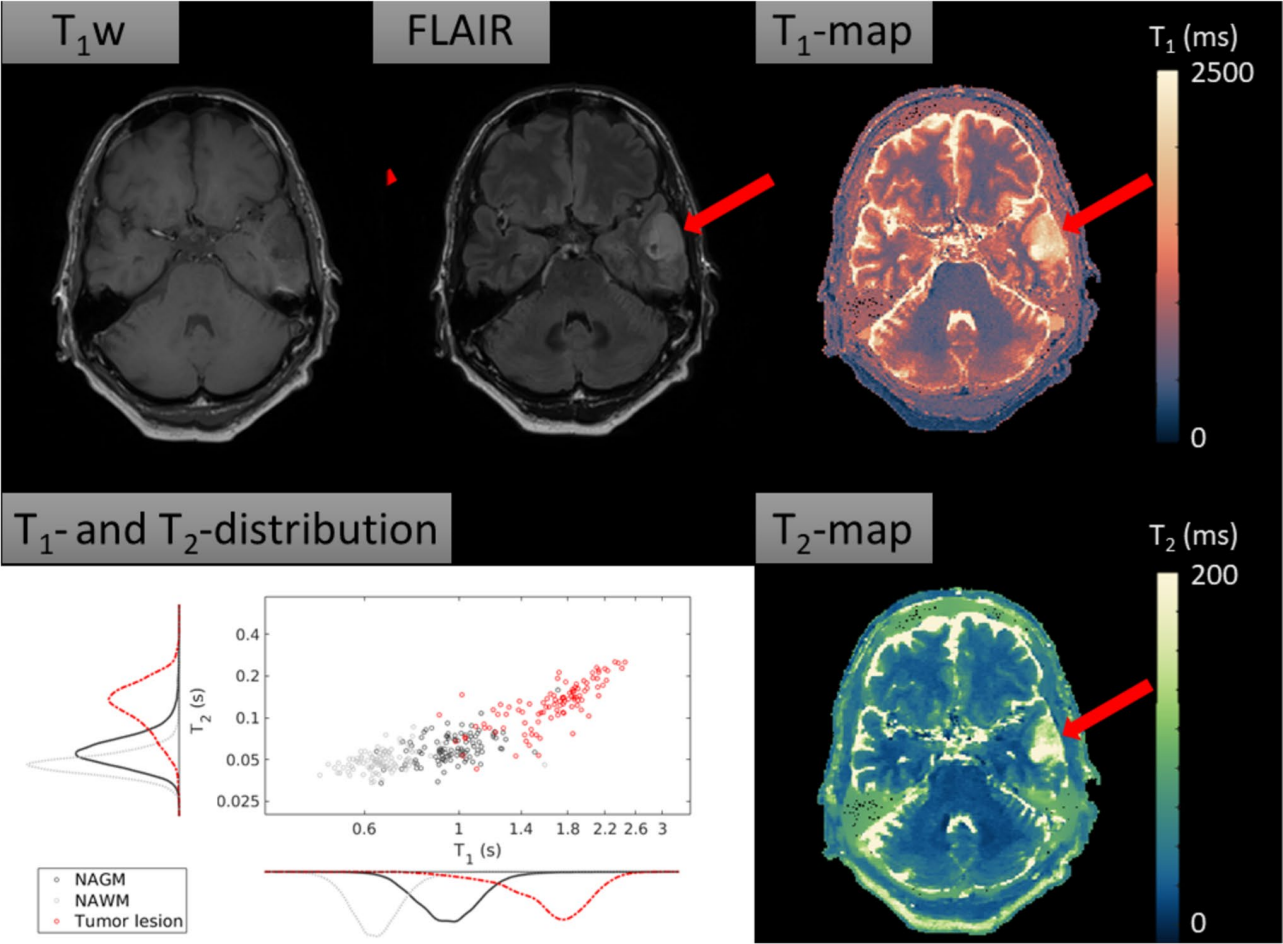
The conventional T_1_w image shows an intra-axial hypointense tumor. The conventional FLAIR image shows a hyperintense tumor. The red arrow denotes the tumor. The *T*_1_-map and the *T*_2_-map show elongation of *T*_1_- and *T*_2_-values in the tumor compared to the surrounding NABTs. Scatterplot and histogram of marginal *T*_1_ and *T*_2_ distributions for each tissue of interest. The quantitative data distribution for the *T*_1_-values and for the *T*_2_-values of the tumor are mostly separable from the NAGM and NAWM. The scatterplot was randomly under-sampled for legibility

Figure 4 shows the conventional T_1_w image, the conventional FLAIR, the *T*_1_-map, the *T*_2_-map and a scatterplot with histograms visualizing the quantitative data distribution for the tumor patient with an anaplastic glioma (WHO 2021 diagnosis: Diffuse glioma grade 3, not elsewhere classifiable). For this patient, the mean *T*_1_ (SD) of the anaplastic glioma (red arrow) was 1729 (353) ms and the mean *T*_2_ (SD) was 140 (53) ms. For the NAGM, the mean *T*_1_- and *T*_2_-values (SD) were 992 (202) ms and 65 (28) ms, respectively, while for the NAWM, the mean *T*_1_- and *T*_2_-values were 661 (116) ms and 50 (19) ms, respectively. The lesion’s *T*_1_ showed an increase of 74.3% and 161.4% compared to NAGM and NAWM, while the lesion’s *T*_2_ showed an increase of 114.4% and 177.8% compared to NAGM and NAWM.

#### Case example 2: Multiple sclerosis

**Fig. 5:**
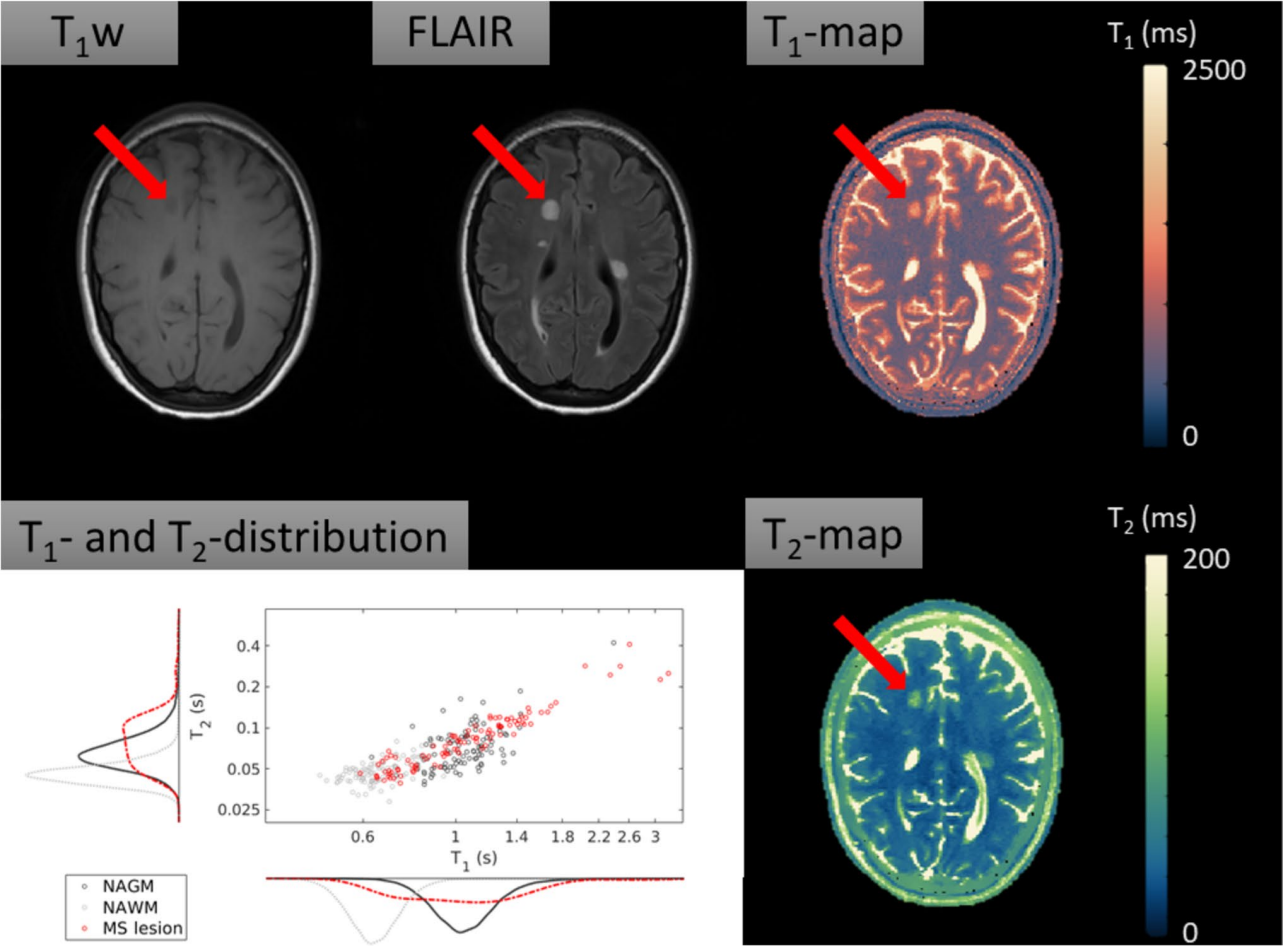
Conventional anatomical images (T_1_w, FLAIR), quantitative maps (*T*_1_, *T*_2_) and a scatterplot with histograms visualizing the quantitative data distributions for each tissue of interest. T_1_w image shows multiple hypointense black-hole lesions, which are typical for MS. FLAIR image shows multiple corresponding hyperintense lesions. The *T*_1_-map shows that the lesions have on average longer *T*_1_ values than the normal appearing surrounding tissue. The *T*_2_-map shows longer a transverse relaxation time of the lesion compared to surrounding NAWM. The scatterplot with quantitative data distribution shows that, compared to the NAWM, the lesion does no longer have similar *T*_1_- and *T*_2_-values The scatterplot was randomly under-sampled for legibility

In the second case, a patient with MS was investigated. Figure 5 shows the corresponding anatomical images (conventional T_1_w, conventional FLAIR), as well as quantitative maps (*T*_1_, *T*_2_) and a scatterplot with histograms visualizing the quantitative data distributions for each tissue of interest. One of the MS lesions has been denoted with a red arrow. For the selected lesion, the mean *T*_1_ (SD) was 1084 (408) ms and the mean *T*_2_ (SD) was 87 (48) ms. Of the NAWM, the mean *T*_1_ was 643 (104) ms and the mean *T*_2_ was 47 (14) ms. This means that the lesion’s *T*_1_ showed an increase of 68.6%, while the lesion’s *T*_2_ showed an increase of 85.1% compared to the *T*_1_ and *T*_2_ of NAWM.

## Discussion

### Summary of main findings

In order to add to the validity of MR-STAT as a clinically applicable fast relaxometry technique, this study sought to report *T*_1_- and *T*_2_-values of NBTs and NABTs acquired in 49 participants. Differences between male and female participants were not statistically significant. Furthermore, statistically significant differences in relaxation times across hemispheres were seen in the parietal lobe and in the temporal lobe. We found a significant quadratic age-dependency of *T*_1_ of the caudate nucleus, *T*_1_ of the globus pallidus, *T*_2_ of the thalamus and *T*_2_ of the globus pallidus. All other quadratic age-dependencies were not statistically significant. Lastly, in two individual case examples, quantitative values of pathological tissues were presented, i.e. for a tumor patient and an MS patient, with clear differences between lesions versus normal tissues.

### Comparison to literature

The relaxation times of N(A)GM and N(A)WM reported in this paper are within ranges of relaxation times at a field strength of 3 T reported in recent literature (*T*_1_: NAGM = 790–1618 ms; NAWM = 620–954 ms; *T*_2_: NAGM = 53–130 ms; NAWM = 29–120 ms) [7, 8, 11, 13, 14, 25, 26, 29–45]. The measured *T*_1_- and *T*_2_-values of thalamus, putamen, caudate nucleus and globus pallidus were lower than or at the lower end of earlier reported ranges (*T*_1_: thalamus = 860–1262 ms; putamen = 920–1328 ms; caudate nucleus = 960–1379 ms; globus pallidus = 800–1055 ms; *T*_2_: thalamus = 60–74 ms; putamen = 49–64 ms; caudate nucleus = 59–73 ms; globus pallidus = not available) [7, 8, 11, 13, 14, 25, 26, 29–45].

Earlier work using MR-STAT relaxometry in gel phantoms presented good correlation with ground truth values [17, 46]. However, in vivo, confounding factors that potentially influence the measured *T*_1_- and *T*_2_-values include the magnetization transfer (MT) effect, subject motion and flow of blood and cerebrospinal fluids. MT effects are not described in the Bloch equations that are used in the MR-STAT framework. However, it was demonstrated in MRF-based experiments that correcting for MT leads to an increase of 12–25% for *T*_1_, and 16–34% for *T*_2_, where the effects of MT corrections was generally stronger in more myelinated tissues [45]. Possibly, not correcting for MT contributed also to the relatively low reported *T*_1_- and *T*_2_-values observed in our study. No motion artefacts were observed and flow related artefacts were confined to the ventricles according to radiological assessment [20]. Consequently, the effect of motion and flow on the presented *T*_1_- and *T*_2_-values of NBTs, NABTs and lesions is likely negligible.

Regarding the differences in relaxation times of NGM and NWM between male and female participants, no statistically significant differences were found. Earlier literature also reported that no statistically significant differences were found [8, 26]. With respect to the left–right brain lobe comparison, we found statistically significant left–right differences in the *T*_1_ of NGM of the temporal lobe, in the *T*_2_ of the NWM of the parietal lobe and the temporal lobe, and in the *T*_2_ of the NWM of the temporal lobe. Earlier literature describes hemispheric asymmetries for the *T*_1_ of NWM of the temporal lobe, the *T*_2_ of NWM of the frontal lobe and the *T*_2_ of NWM of the parietal lobe [6]. The statistically significant differences we found could be attributable to microstructural differences that originate from unilateral dominance for language, lateralization of spatial recognition and WM connectivity [47]. Concerning the differences between brain lobes, the differences were not statistically significant for the *T*_1_ of NGM and NWM.

Age-dependencies of *T*_1_- and *T*_2_-values of the N(A)GM, N(A)WM, thalamus, putamen, caudate nucleus and globus pallidus were investigated. Using an established quadratic model [26], we found that the age-dependencies for *T*_1_-values of the caudate nucleus and the globus pallidus, and the *T*_2_-values of the thalamus and the globus pallidus were statistically significant. Similar results were found in literature for the age-dependency of *T*_1_-values of the caudate nucleus [26], *T*_1_-values of the globus pallidus [25] and the *T*_2_-values of the thalamus [26]. However, the relationship between age and *T*_2_-values for the globus pallidus was not addressed in recent literature. Although the quadratic relationships were not significant for all brain regions, a quadratic trend was observed in our data, similar to earlier literature [6, 25, 26]. Factors that contribute to these quadratic models for relaxation times include non-pathological age-related loss of myelination, increased free water content and ion depositions [6, 25, 26]. However, in the current analysis, the potential influence of disease progression on relaxation times of NABTs cannot be excluded and literature on this topic is scarce. Also, there is no consensus in literature on whether to use a linear fit, a quadratic fit or a more complex fit to model age-dependencies of relaxation times of brain regions, nor is there consensus on whether this differs per brain region. However, the strong relationship between age and relaxation times underlines the importance of age when considering normative *T*_1_- and *T*_2_-values for N(A)BTs.

With respect to the case examples, we reported a mean *T*_1_ of 1729 (± 353) ms and a mean *T*_2_ of 140 (± 53) ms for the grade 3 anaplastic glioma. Springer et al*.* reported average *T*_1_-values ranging between 1770 and 2068 ms, and average *T*_2_-values ranging between 79 and 144 ms for grade 3 glioma [48], which fall within one standard deviation from our measurements. Regarding the multiple sclerosis case study, the lesion in our patient showed an increase of 68.6% in *T*_1_-values and increase of 85.1% in *T*_2_-values when compared to the quantitative values of NAWM. The increased *T*_1_- and *T*_2_-values can be explained by the inflammatory and demyelinating brain pathology in MS patients [49]. At 1.5 T [50], the reported *T*_1_-values of enhancing and non-enhancing lesions were on average 40.2% and 92.1% higher than the average *T*_1_-values of NAWM. Reported *T*_2_-values of enhancing and non-enhancing lesions were on average 33.7% and 77.0% higher than the *T*_2_-values of NAWM for the study population as a whole. A direct comparison of relaxation times was not possible, since the studies were performed at different field strengths and the enhancement status of our patient’s lesion is unknown. Yet, the percentage wise increase confirms characteristics of MS lesions described in earlier literature. Overall, in diseased tissues, similar trends were observed in *T*_1_- and *T*_2_-values measured with MR-STAT and *T*_1_- and *T*_2_-values reported in literature. This offers preliminary support for the clinical validity of MR-STAT as fast relaxometry protocol.

### Limitations

Firstly, statistical tests 1–3 necessitate cautious interpretation. Only data from young, healthy participants was used, and the results might therefore lack generalizability for an elderly population. Moreover, we compared male healthy volunteers (*n* = 6) against female healthy volunteers (*n* = 4) in test 1. Due to relatively low number, the robustness of this test is limited. We believe the validity of tests 2 and 3 (*n* = 10) remains reasonably strong, indicating there is no systematic left/right bias and thus preliminarily supporting the clinical utility of MR-STAT.

Secondly, our study population is heterogeneous in terms of age, sex and clinical status. Also, despite our efforts to exclude all pathological tissue from the statistical tests, it is still possible that some of this tissue was erroneously classified as NABTs. In particular, we are aware that MS patients can have elevated *T*_2_-values (approximately 2 ms higher) in the parietal and temporal NAWM [51]. However, in the current study, patient data from NABTs were used in the age-dependency analysis only and the trends observed in this analysis aligned with literature.

Thirdly, due to the spatial resolution of the current sequence, data might be subject to partial volume effects (PVE) or relevant tissues might be partially missing. We observed that we measured *T*_1_- and *T*_2_-values in subcortical brain regions that were lower than or at the lower end of values in literature. Potentially, the mean relaxation times of the subcortical brain regions have been offset by surrounding NAWM that might have contaminated the corresponding masks. Furthermore, the PVE might be reflected in the span of *T*_1_- and *T*_2_-values in the N(A)GM, which are globally wider than the *T*_1_- and *T*_2_-values of N(A)WM as an effect of CSF contamination of the N(A)GM mask (Fig. 2). Locally, a relatively wide span in *T*_1_-values of N(A)GM of the occipital lobe is observed compared to the other brain lobes. As the N(A)GM of the occipital lobe is thinner compared to the N(A)GM of the other brain lobes [52], more PVE might occur. An example of a relevant missing subcortical nucleus is the substantia nigra, which is an important region to image in the diagnosis of Parkinson’s disease [53]. To alleviate PVE and to ensure no relevant tissues are missing, future work should focus on creating a 2D MR-STAT sequence with isotropic resolution without interslice gaps, or on creating a 3D MR-STAT sequence [22, 23].

Lastly, since we compared multiple variables in each statistical test, we performed Bonferroni correction extensively. This reduces the chance of finding statistically significant differences, however, at the cost of increased chance of making type I errors. Some findings may thus incorrectly have been regarded as not statistically significant.

### Clinical relevance

The reported relaxometry values could be used as reference values for NBTs and NABTs in future research that uses this sequence. In the case examples we provided, diseased tissue could clearly be discerned from NABTs using quantitative values. This opens up possibilities for voxel based clustering and other automated classification and segmentation methods in the future. We would like to stress that more research with clearly defined, sufficiently sized cohorts is needed to prove the value of MR-STAT in addition to or as substitute of conventional MRI.

### Future outlook

Whereas the current work focused on NBTs and NABTs, future work involving MR-STAT should shift its attention to finding added value to clinical routine. One possible direction is to leverage the values from the quantitative parameter maps for data science and machine learning purposes, such as tumor classification or anomaly detection. One publication describes the use of MRF to differentiate between two subtypes of glioma by analyzing the quantitative values of the solid parts of the tumors, the peritumoral edema and the NAWM [48]. In another study, methods to build voxel-wise quantitative brain relaxation atlases are described. The quantitative relaxation atlases are then used to compute a voxel-wise statistical deviation map. Using these deviation maps, MS lesions could clearly be identified from NABTs [54]. Potentially, these applications could help guide clinicians in their decision-making with the support of automated data-analysis tools.

## Conclusion

In this study, relaxation times and data trends of NBTs and NABTs using MR-STAT data of 49 subjects were reported for the first time. Furthermore, two individual clinical cases were presented using MR-STAT in a clinical context. Since our findings were generally in line with earlier relaxometry literature, this work preliminarily adds to the validity of MR-STAT as a clinically feasible option for fast relaxometry. More research is needed to define, improve and validate its clinical value.

## Supplementary Information

Below is the link to the electronic supplementary material.

Supplementary file1 (DOCX 81 KB)

## Data Availability

Example images of the quantitative parameter maps (*T*_1_, *T*_2_, PD) and conventional and synthetic contrast weighted images including all 30 slices from a healthy participant and a patient of each group are in a publicly available repository (https://gitlab.com/asbrizzi/mr-stat-synthetic-images).

## Abbreviations

CN: Caudate nucleus
FLAIR: Fluid attenuated inversion recovery
GP: Globus pallidus
MR-STAT: Magnetic Resonance Spin TomogrAphy in Time-domain
MRF: Magnetic Resonance Fingerprinting
MS: Multiple sclerosis
NABT: Normal appearing brain tissue
NAGM: Normal appearing grey matter
NAWM: Normal appearing white matter
NBT: Normal brain tissue
NGM: Normal grey matter
NWM: Normal white matter
PD: Proton density
PVE: Partial volume effect
QRAPTEST: Quantification of relaxation times and proton density by twin-echo saturation-recovery turbo-field echo
STAGE: STrategically Acquired Gradient Echo
syMRI: Synthetic magnetic resonance imaging
TrueFISP: True fast imaging with steady state precession
