# Supplementary material for: Fast whole brain relaxometry with Magnetic Resonance Spin TomogrAphy in Time-domain (MR-STAT) at 3 T: a retrospective cohort study
Source: MAGMA. 2025 Mar 4;38(2):333–45. doi: 10.1007/s10334-025-01237-3 (PMC11914305; doi:10.1007/s10334-025-01237-3)
Supplement: Supplementary file 1 — Supplementary file1 (DOCX 81 KB) [file 10334_2025_1237_MOESM1_ESM.docx]

# Supplementary Material A


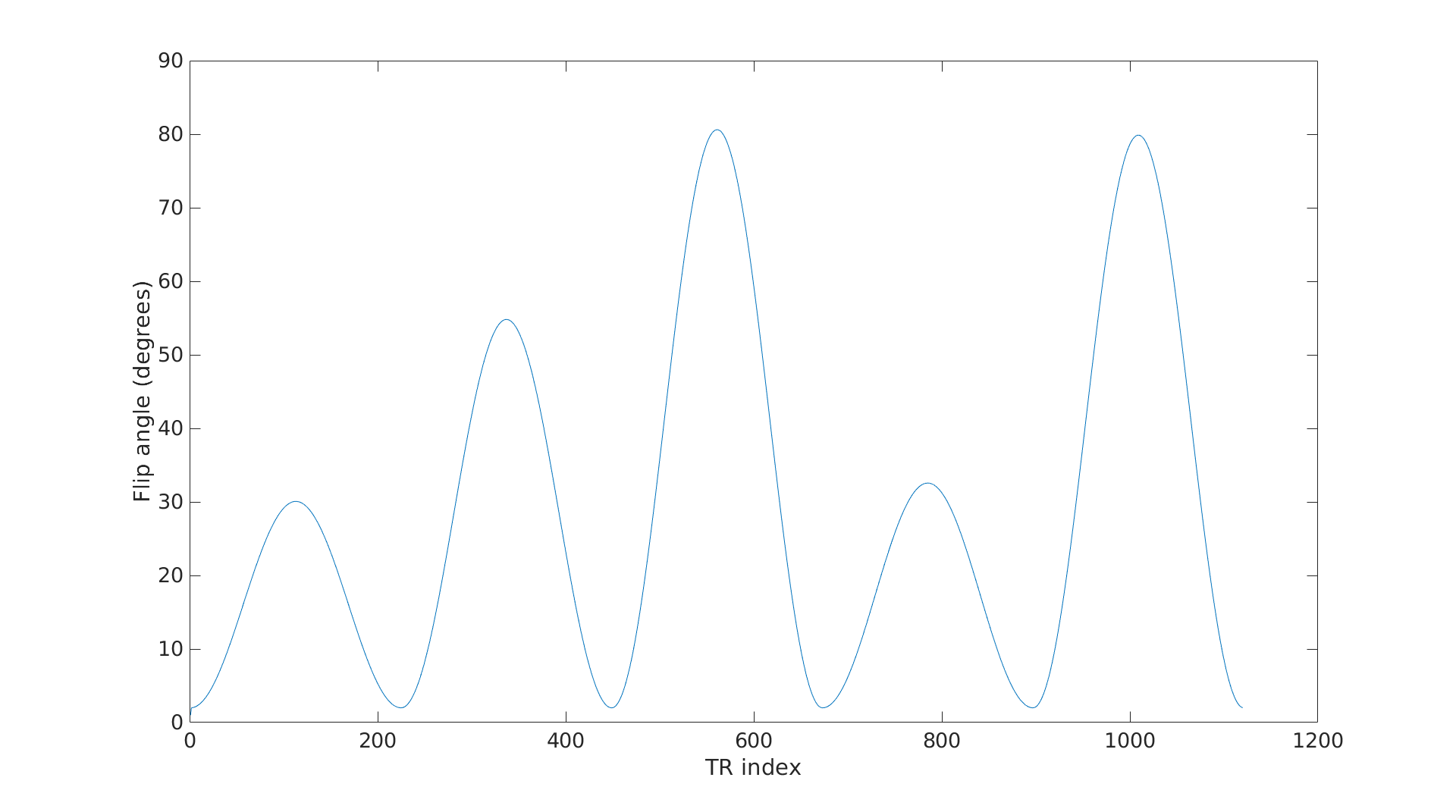
The flip angle profile is shown in the figure below.

# Supplementary Material B

Mean relaxation times (standard deviation) per clinical group.

|  | Tumor patients without radiotherapy | Tumor patients with radiotherapy | Stroke patients | Epilepsy patients | MS patients |
| --- | --- | --- | --- | --- | --- |
| T_1_ NAGM | 1087 (104) ms | 1064 (84) ms | 1067 (57) ms | 1070 (30) ms | 1091 (58) ms |
| T_1_ NAWM | 653 (16) ms | 658 (26) ms | 673 (30) ms | 640 (16) ms | 661 (31) ms |
| T_1_ Thalamus | 765 (25) ms | 788 (78) ms | 784 (36) ms | 783 (60) ms | 798 (31) ms |
| T_1_ Putamen | 857 (20) ms | 843 (38) ms | 871 (47) ms | 868 (39) ms | 856 (31) ms |
| T_1_ Caudate nucleus | 997 (54) ms | 1039 (119) ms | 1050 (119) ms | 1040 (103) ms | 1073 (93) ms |
| T_1_ Globus pallidus | 636 (17) ms | 645 (49) ms | 642 (37) ms | 651 (32) ms | 659 (39) ms |
| T_2_ NAGM | 73 (9) ms | 72 (12) ms | 73 (9) ms | 73 (3) ms | 76 (8) ms |
| T_2_ NAWM | 48 (2) ms | 49 (5) ms | 50 (4) ms | 49 (1) ms | 50 (2) ms |
| T_2_ Thalamus | 40 (2) ms | 44 (7) ms | 42 (3) ms | 42 (2) ms | 42 (3) ms |
| T_2_ Putamen | 44 (1) ms | 45 (4) ms | 47 (5) ms | 45 (2) ms | 47 (3) ms |
| T_2_ Caudate nucleus | 58 (4) ms | 62 (10) ms | 66 (13) ms | 61 (7) ms | 66 (9) ms |
| T_2_ Globus pallidus | 33 (2) ms | 38 (9) ms | 36 (3) ms | 34 (2) ms | 36 (3) ms |

# Supplementary Material C

P-values from Wilcoxon sign rank test, comparing between brain lobes (test 2). **Bold** indicates statistical significance.

|  | Frontal vs Temporal | Frontal vs Parietal | Frontal vs Occipital | Temporal vs Parietal | Temporal vs Occipital | Parietal vs Occipital |
| --- | --- | --- | --- | --- | --- | --- |
| T_1_ NAGM | 0.2754 | 0.0371 | 0.6953 | 0.4316 | 0.0098 | 0.0371 |
| T_1_ NAWM | 0.9219 | 0.6250 | 0.8457 | 0.7695 | 0.9219 | 1.0 |

# Supplementary Material D

P-values from Wilcoxon sign rank test, comparing across left and right brain lobes (test 3). **Bold** indicates statistical significance.

|  | Frontal left vs right | Temporal left vs right | Parietal left vs right | Occipital left vs right |
| --- | --- | --- | --- | --- |
| T_1_ NAGM | 0.4316 | **0.0020** | **0.0020** | 0.1055 |
| T_1_ NAWM | 0.0137 | 0.0840 | **0.0020** | 0.3223 |
| T_2_ NAGM | 0.3750 | 0.3223 | 0.8457 | 0.4922 |
| T_2_ NAWM | 0.0273 | **0.0020** | 0.8457 | 0.5566 |

# Supplementary Material E

Mean (sd) T_1_ and T_2_ relaxation times of brain lobes of healthy volunteers

|  | Left frontal lobe | Right frontal lobe | Left temporal lobe | Right temporal lobe | Left parietal lobe | Right parietal lobe | Left occipital lobe | Right occipital lobe |
| --- | --- | --- | --- | --- | --- | --- | --- | --- |
| T_1_ NAGM | 1118 (70) ms | 1117 (72) ms | 1136  (95) ms | 1151 (104) ms | 1137 (86) ms | 1162 (90) ms | 1102 (117) ms | 1114 (111) ms |
| T_1_ NAWM | 670  (20) ms | 664  (20) ms | 662  (24) ms | 667  (23) ms | 662  (24) ms | 662  (14) ms | 657  (29) ms | 669  (38) ms |
| T_2_ NAGM | 87  (16) ms | 86  (13) ms | 72  (11) ms | 71  (11) ms | 78  (15) ms | 79  (15) ms | 70  (15) ms | 69  (15) ms |
| T_2_ NAWM | 55  (3) ms | 54  (3) ms | 46  (3) ms | 44  (3) ms | 48  (3) ms | 48  (4) ms | 44  (4) ms | 45  (5) ms |

# Supplementary Material F

Table with coefficients of determination, p-values for quadratic fit and curve coefficients. **Bold** indicates statistical significance.

|  | R^2^ | p-value | Intercept | Linear coefficient | Quadratic coefficient | Minimum of curve (years) |
| --- | --- | --- | --- | --- | --- | --- |
| T_1_ NAGM | 0.124 | 0.0482 | 1291 | -9.295 | 0.932 | 50 |
| T_1_ NAWM | 0.196 | 0.0066 | 684 | -1.901 | 0.027 | 35 |
| T_1_ Thalamus | 0.216 | 0.00503 | 931 | -7.961 | 0.093 | 43 |
| T_1_ Putamen | 0.147 | 0.0257 | 988 | -5.715 | 0.006 | 49 |
| T_1_ Caudate nucleus | 0.289 | **0.00039** | 1350 | -16.91 | 0.200 | 42 |
| T_1_ Globus pallidus | 0.290 | **0.00037** | 802 | -7.383 | 0.080 | 46 |
| T_2_ NAGM | 0.136 | 0.0348 | 101 | -1.281 | 0.0133 | 48 |
| T_2_ NAWM | 0.146 | 0.0267 | 55 | -0.365 | 0.0044 | 41 |
| T_2_ Thalamus | 0.286 | **0.00044** | 49 | -0.463 | 0.006 | 37 |
| T_2_ Putamen | 0.118 | 0.0555 | 55 | -0.420 | 0.0004 | 49 |
| T_2_ Caudate nucleus | 0.148 | 0.0255 | 83 | -1.131 | 0.0014 | 41 |
| T_2_ Globus pallidus | 0.223 | **0.003** | 43 | -0.4 | 0.0048 | 43 |
